# Response Surface Methodology for Boron Removal by Donnan Dialysis: Doehlert Experimental Design

**DOI:** 10.3390/membranes11100731

**Published:** 2021-09-25

**Authors:** Ikhlass Marzouk Trifi, Lobna Chaabane, Lasâad Dammak, Lassaad Baklouti, Béchir Hamrouni

**Affiliations:** 1 Laboratoire de Recherche Dessalement et Traitement des Eaux, Faculté des Sciences de Tunis, Université de Tunis El Manar, Tunis 1068, Tunisia; ikhlassmarzouk@gmail.com (I.M.T.); bechir.hamrouni@fst.utm.tn (B.H.); 2 Institut de Chimie et des Matériaux Paris-Est (ICMPE), Université Paris-Est, UMR 7182, CNRS, 2-8 rue Henri Dunant, 94320 Thiais, France; lobna.dammak@u-pec.fr; 3 Department of Chemistry, College of Sciences and Arts at Al Rass, Qassim University, Ar Rass 51921, Saudi Arabia; bakloutilassaad@yahoo.fr

**Keywords:** boron, Doehlert design, Donnan dialysis, optimization, response surface methodology

## Abstract

The removal of boron by Donnan dialysis from aqueous solutions has been studied according to response surface methodology (RSM). First, a preliminary study was performed with two membranes (AFN and ACS) in order to determine the experimental field based on different parameters, such as the pH of the feed compartment, the concentration of counter-ions in the receiver compartment, and the concentration of boron in the feed compartment. The best removal rate of boron was 75% with the AFN membrane, but only 48% with the ACS membrane. Then, a full-factor design was developed to determine the influence of these parameters and their interactions on the removal of boron by Donnan dialysis. The pH of the feed compartment was found to be the most important parameter. The RSM was applied according to the Doehlert model to determine the optimum conditions ([B] = 66 mg/L, pH = 11.6 and [Cl^−^] = 0.5 mol/L) leading to 88.8% of boron removal with an AFN membrane. The use of the RSM can be considered a good solution to determine the optimum condition for 13.8% compared to the traditional “one-at-a-time” method.

## 1. Introduction

Water present in nature may often neither be used for human consumption nor for industry use, because it may contain relatively high concentrations of various chemical compounds such as boron. There is a small range between boron deficiency and boron toxicity in vegetable crops and other plants [1]. Boron is an essential nutrient for plants; a very low boron content is required in irrigation water for certain metabolic activities, but an increased boron concentration to 4 mg/L [1] leads to poisoning, reflected by yellowish spots on leaves and fruits, and accelerates decomposition and kills the plants [2]. In drinking water, high boron concentrations can be also toxic to humans. The WHO indicate that the concentration of boron in drinking water should be less than 0.3 mg/L [3]. The boron concentration is around 5 mg/L in seawater [4]. The growth in knowledge of the science of boron in recent years has been of great commercial and environmental importance because the element is used in a wide range of industrial applications such as in insulation and textile-grade fiber, borosilicate glass, fire retardants, enamels, glazes and agriculture. The presence of boron in water originates from different sources, such as mineralization. In urban wastewater systems, boron is mainly found in the form of boric acid or borates. Boron removal has been investigated using adsorption [5,6], electrocoagulation [7,8], electrodialysis [9,10], reverse osmosis [11,12], nanofiltration [13,14], microfiltration [15], ion-exchange [15,16], membrane distillation [17] and Donnan dialysis [18,19]. In order to choose the simplest and continuous method, Donnan dialysis was conducted in this study. This process is easy to handle and only requires a few chemicals and low pumping energy. Donnan dialysis is widely used to recover or concentrate [20,21,22] and eliminate various ions such as nitrates [23], nitrates and nitrites simultaneously [24], fluorides [25,26,27], chromium [28,29], phosphates [30] and boron [18,19].

Herein, the removal of boron by Donnan dialysis was investigated using a response surface methodology (RSM) approach. RSM is a collection of mathematical and statistical techniques based on the fit of a polynomial equation to the experimental data, which must describe the behavior of a dataset with the objective of making statistical previsions. The advantage of the RSM approach for optimization is the simultaneous variation of all factors at once in each experiment, the decrease in the number of attempts, the study of a large number of factors, the detection of interactions between factors and obtaining the best possible precision [31]. RSM via the Doehlert design was employed in this study. This design offers many advantages over other designs such as central composite design or Box–Behnken design. Doehlert designs involve a reduced number of experiments. All variables have different numbers of levels, which allows flexibility to assign a large or a small number of levels to the selected variables. In order to obtain maximum information of the system, it is preferable to choose the variable with the stronger effect as the variable with maximum levels. Finally, considering that the efficiency of any experimental design is defined as the number of coefficients of the model divided by the number of experiments, Doehlert design is more efficient than central composite design or Box–Behnken design [24].

In this study, first, the removal of boron was performed with four parameters: counter-ion concentration in the receiver compartment, boron concentration, the pH in the feed compartment, and anionic exchange membrane type. Secondly, a full factorial design was utilized to study the parameter effects and their interactions. Finally, response surface methodology using Doehlert design was investigated to optimize the Donnan dialysis process.

## 2. Experimental

### 2.1. Membranes

Two membranes were used in the Donnan dialysis process, which were Neosepta^®^ AFN and Neosepta^®^ ACS (supplied by Alstom, Saint-Ouen, France). Their properties were determined according to a French standard NF X 45–200 [32] and are listed in Table 1. The ion-exchange capacity (in meq. of functional sites per gram of dry membrane or per cm^3^ of wet membrane) was determined following the French standard NF X 45-200 [32].

The water content was determinate with a Mettler-Toledo moisture thermo balance device. The water content was calculated with the following relationship:

$$\mathrm{WC}\left(\%\right)=\frac{W_{h}{-W}_{d}}{W_{h}}\;\times 100$$

where WC% is the water content percentage, W_h_ is the hydrated membrane mass, and W_d_ is the dried membrane mass. The water content is the mass difference between the hydrated membrane (immersed in the appropriate stabilization content and pressed slightly in order to remove the excess liquid), and the dried membrane at 140 °C (until membrane mass stabilization indicated the total removal of water).

The membrane thickness in the dry state corresponded to a mean value of 10 measurements at different locations using a 1 μm resolution Käfer Thickness Dial Gauge.

Conditioning of the samples before any measurement was necessary, essentially to remove impurities coming from the manufacturing process, as well as to stabilize their physical–chemical properties in order to prepare the samples for use in Donnan dialysis. This conditioning performed done according to the French standard NF X 45–200 [32].

### 2.2. Donnan Dialysis (DD)

Donnan dialysis is a membrane separation process in which an exchange of ions having the same electrical charge between two solutions through an ion-exchange membrane takes place [33,34,35]. The driving force in Donnan dialysis is the chemical potential gradient; there is a stoichiometric exchange of anions through an anionic exchange membrane, and the process ends only when the Donnan equilibrium is reached. Electroneutrality is sustained; therefore, the same amounts of anions should be exchanged from the feed to the receiver compartment, and vice versa [19,36,37].

**Figure 1:**
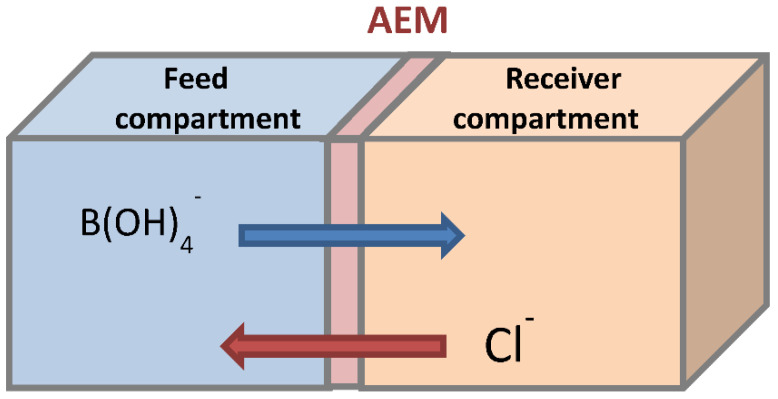
Schematic flow diagram of a Donnan dialysis system for boron removal (in an alkaline environment).

Figure 1 shows a schematic flow diagram of Donnan dialysis. The feed and receiver tanks Erlenmeyer flask were used to supply the two cell compartments using a controlled peristaltic pump. In the receiver compartment, solutions containing NaCl were prepared, and in the feed compartment, a solution containing boron was prepared. The membrane was sandwiched between these two compartments, making a seal at the same time. Two membranes, AFN and ACS, were used. Each experiment lasted seven hours. During dialysis operation, different effects were investigated in order to determine the concentrations of boron. The samples were analyzed for boron concentration by reactions with azomethine-H followed by absorbance measurements at 420 nm using a UV–visible spectrophotometer [38]. The concentration was linear in the range of 1–4 mg/L. The samples of higher concentration were diluted in order to match the above linearity range.

The removal rate of boron was calculated with Equation (1):

(1) $$Y\left(\%\right)=\frac{C_{0}{-C}_{e}}{C_{0}}\;\times \;100\;$$

where C_0_ is the initial boron concentration (mg/L), and C_e_ is the equilibrium boron concentration (mg/L).

### 2.3. Optimization of the Removal of Boron by Donnan Dialysis

First, a full factorial design was used in order to determine the most important and influential parameter in the RSM design according to Doehlert matrix. The software used in this study was NemrodW^®^. NemrodW^®^ software was an essential support for the practical implementation of the experimental research methodology (experimental designs).

## 3. Results and Discussion

### 3.1. The Preliminary Study

#### 3.1.1. Effect of pH in the Feed Compartment

**Figure 2:**
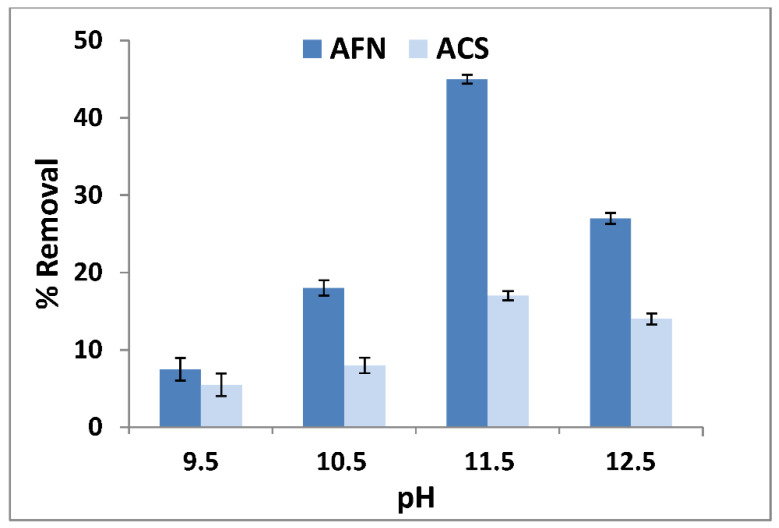
pH effects on the removal rate of boron.

The pH effect in the feed compartment was determined with different initial pH values of feed solutions, ranging from 9.5 to 12.5, with a concentration of counter-ion of 0.1 mol/L, initial boron concentration of 50 mg/L and stirring speed of 500 rpm. The two membranes, AFN and ACS, were tested for dialysis operations lasting seven hours. At the outlet of the receiver solution, the variations in boron concentrations under different initial pH values are presented in Figure 2.

The effect of pH was studied first, due to its impact on the transport of boron from the feed compartment to receiver compartment. As shown in Figure 2, the highest removal of boron was obtained at a pH of 11.5, with 45 ± 0.6% for the AFN membrane and 17 ± 0.6% for the ACS membrane. This can be explained by the two existing forms of boron species in aqueous solution under different pH values, which were boric acid B(OH)_3_ in dilute aqueous solution below pH 7, and above pH 10, the metaborate anion ${\;B(\mathrm{OH})}_{4}^{-}$ became the dominant species in solution [39,40]. However, above pH = 11.5, the transport of boron was probably affected by the presence and competition with OH^−^, which decreased boron removal because hydroxyl ion transport was preferred, because the mobility of OH^−^ is much higher than that of boron. In fact, the transport of boron is a process which involves three steps: the boron in feed solution is exchanged with ions or ionizable groups in an anion-exchange membrane; boron is transported in a membrane to the side of the receiver solution; and boron is transferred to a receiver solution with counter ions [21,26]. Thus, it may be concluded that the highest boron transport was achieved at pH = 11.5 for the two used membranes.

#### 3.1.2. Effect of Chloride Concentration in the Receiver Compartment

**Figure 3:**
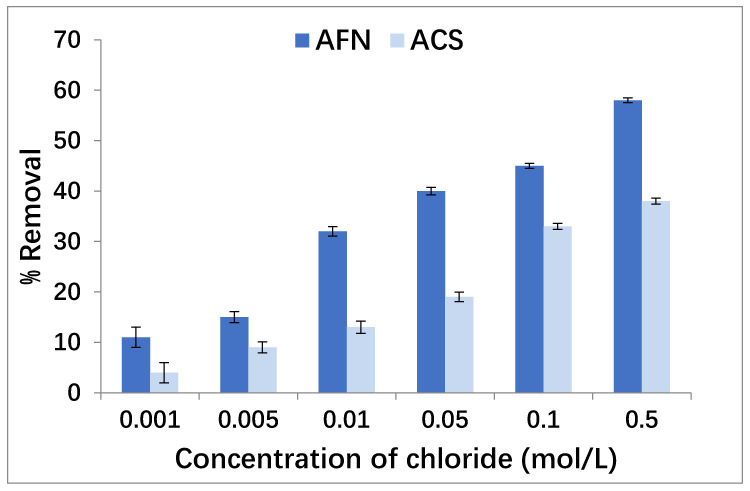
Effect of chloride concentration in the receiver compartment (pH 11.5, boron concentration 50 mg/L in the feed compartment, stirring speed of 500 rpm and temperature 25 °C).

The chloride concentration is one of the parameters affecting the removal of boron through the anionic exchange membrane during the Donnan dialysis operation. Variation in the concentration of counter-ion Cl^−^ from 0.001 mol/L to 0.5 mol/L in the receiver compartment was investigated in order to explain this impact on the transport of boron (50 mg/L) through the anionic exchange membrane from the feed compartment to the receiver compartment. The effects of chloride concentration in the receiver compartment for the two membranes are presented in Figure 3, with a pH of 11.5, a boron concentration of 50 mg/L in the feed compartment, a stirring speed of 500 rpm and a temperature equal to 25 °C.

Figure 3 suggests that the flux of boron ions through anion exchange membranes increases with the increase in chloride concentration from 0.001 to 0.5 mol/L. The transport of boron was very low at a concentration of 0.001 mol/L of Cl^−^, where the removal efficiency was 11 ± 2% for AFN and 4 ± 2% for ACS. However, the removal efficiency improved to 58 ± 0.47% for AFN and 38 ± 0.47% for ACS at 0.5 mol/L of the concentration of Cl^−^. This is explained by the fact that the concentration gradient of the counter-ions increased; hence, the cross-ion transfer between Cl^−^ and boron improved in order to maintain the electroneutrality. For the two membranes, it seems that the increase in the concentration of counter-ions in the receiver compartment is associated with improvements in boron removal in the feed compartment; this is reflected by improving the kinetics of the exchange. In fact, it is known that ion exchange is faster when the concentration of counter-ions is higher in the receiver compartment [41,42,43].

#### 3.1.3. Effect of Boron Concentration in the Feed Compartment

**Figure 4:**
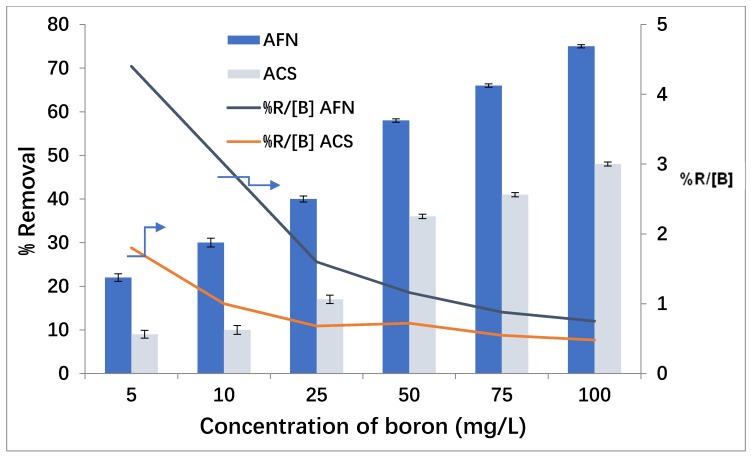
Effect of boron concentration in the feed compartment.

The boron concentration in feed compartment has an important influence on the removal by Donnan dialysis. Under the conditions of pH 11.5 in the feed compartment, the boron concentration increase from 5 mg/L to 100 mg/L, and in the receiver compartment, the Cl^−^ concentration is 0.5 mol/L. The results are presented in Figure 4.

As presented in Figure 4, the increase in initial concentration of boron from 5 mg/L to 100 mg/L contributes to maintaining the high concentration gradient of boron, involving an improvement in the cross-ion transfer between Cl^−^ and ${B(\mathrm{OH})}_{4}^{-}$. At the lowest concentration of boron in the feed compartment (5 mg/L), the removal was 22 ± 1% for AFN and 9 ± 1% for ACS. The increase in boron concentration from 10 mg/L to 100 mg/L improved their removal from 30 ± 1% to 75 ± 0.37% with AFN and from 10 ± 1% to 48 ± 0.5% with ACS. A similar result was reported by Tor [44].

The %R/[B] ratio, expressed as %/(mg/L), indicates the efficiency of boron removal from the feed compartment, in order to explain the diffusion of boron through the membrane. At low concentrations, the diffusion of B through the membrane was performed with the cross-exchange of Cl^−^. However, in high concentrations (of B and/or of Cl^−^), the membrane loses part of its performance (selectivity), and Cl^−^ ions leak without any exchange with boron ions. Therefore, the %R/[B] ratio allows us to conclude that, as the boron concentration in the feed increases, more boron is removed, but in a less efficient manner. As indicated in Figure 4, the %R/[B] ratio: (i) was always higher for the AFN membrane than for the ACS; and (ii) decreased very rapidly and then tended towards an asymptotic value when the initial feed boron concentration increased. For example, for the AFN membrane, at a boron concentration of 5 mg/L, an efficiency of 4.5%/(mg/L) was observed, whereas it was only 0.78%/(mg/L) for a concentration of 100 mg/L.

#### 3.1.4. Membrane Choice

According to the previous results in Figure 3 and Figure 4, it was observed that the efficiency of boron removal by Donnan dialysis depended significantly on the AEM properties. It is clear to that the AFN membrane had the best removal rate of boron, 75 ± 1% in seven hours, compared to the ACS membrane, which had a rate of 48 ± 0.5%. Thus, AFN is the most efficient membrane. Akretche indicated that (i) a high exchange capacity increases the selectivity between monovalent and multivalent anions because of the higher repulsion charge; (ii) a high thickness decreases the diffusion, giving rise to lower ion flux, and (iii) a high water content can decrease the permselectivity and favors the penetration of bulky ions [45]. In fact, the AFN membrane presents a higher permeability to monovalent than bivalent anions with high ion-exchange capacity and the highest water content. On the other hand, the ACS had high thickness and low water content; therefore, it had the lowest permeability. This result is confirmed by the work of Ayyildiz [40], who reported that the removal of boron by Donnan dialysis is more effective with an AFN membrane. Therefore, the AFN membrane was selected for the next study.

### 3.2. Full Factorial Design

The full factorial design 2^k^ was carried out to determine the influence of these factors and their interaction on the removal of boron by Donnan dialysis. The preliminary study (Section 3.1) enabled us to define the experimental field and to determine the level that must take every factor. The three chosen factors were the initial concentration of boron, the concentration of counter-ions, and the pH of the feed compartment. The choice of limits was fixed in order to better define the studied response (boron removal efficiency). The Donnan dialysis operated with the AFN membrane.

In order to evaluate the influence of operating parameters on the removal of boron by Donnan dialysis, full factorial design was performed. In Table 2, experimental ranges and factors level are presented. In the present study, for the three factor designs mentioned, a full factorial matrix consisting of a set of eight different experiments was used. The experimental response associated with this factorial design is represented by a linear polynomial model, taking into account the interactions between two parameters (second-order model) and neglecting the third-order interactions (X_1 × 2_X_3_) considered null (Equation (2)):

Y = b_0_ +b_1_X_1_ + b_2_X_2_ + b_3_X_3_ + b_12_X_1_X_2_ +b_13_X_1_X_3_ + b_23_X_2_X_3_ (2)

where Y is the experimental response, X_i_ is a coded variable, b_i_ is an estimation of the principal effect of factor i for the response Y, and b_ij_ is an estimation of the interaction effect between factor i and j for the response Y.

According to the obtained results in Table 3, the coefficients of the model were calculated, and it was found that (Equation (3)):

Y(%) = 34.85 + 2.50 X_1_ + 3.75 X_2_ + 6.15 X_3_ − 0.20 X_1_X_2_ − 0.90 X_1_X_3_ − 1.15 X_2_X_3_ (3)

**Figure 5:**
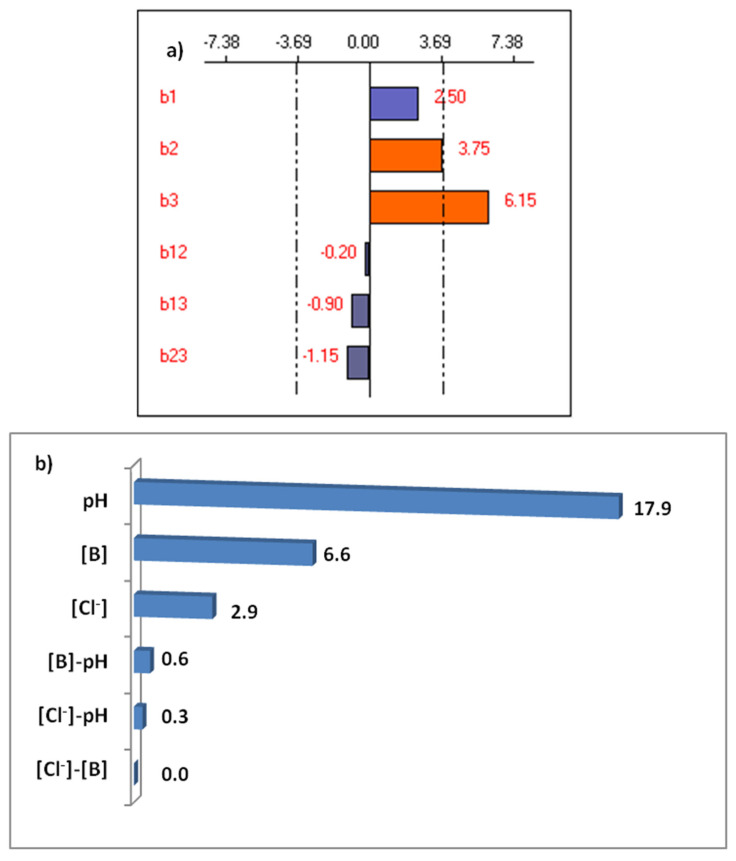
(**a**) Graphical analysis of the removal of boron, (**b**) Pareto effect.

The last column of Table 3 represents the absolute relative differences (%) between the experimental and the calculated values. We note that these differences are very small and do not exceed 1.50%. The different coefficients of the polynomial model (Equation (3); R^2^ = 0.999), representing the effects and interactions of the various investigated factors, are shown in Figure 5a. The Pareto analysis (Figure 5b) allowed us to check the weight of different coefficients in the experimental domain investigated [46]. Thus, this analysis was calculated using Equation (4):

(4) $$P_{i}={\left(\frac{b_{i}^{2}}{\sum b_{i}^{2}}\right)}^{2}\times 100\;$$

The three studied parameters had a positive effect on the studied response, i.e., increasing them led to improvements in the boron removal. Their contributions to the studied response were only 6.6% for boron concentration and 2.9% for chloride concentration versus 17.9% for pH. Thus, boron removal can be considerably influenced by two parameters: pH and boron concentration. The positive sign of the coefficient for the solution pH means that boron removal was improved. This is due to the presence of ${B(\mathrm{OH})}_{4}^{-}$, which becomes the dominant species at high pH values. The counter-ion concentration and the boron concentration have moderately significant effects on the removal of boron by Donnan dialysis. Thus, the most important parameter is the pH of the feed compartment.

### 3.3. Response Surface Methodology

#### 3.3.1. Doehlert Design

The response surface methodology (RSM) according to Doehlert design was performed in this study in order to determine the optimal condition. Doehlert’s approach is formed by uniformly distributing the experimental points within the space filling the variables. The number of experiments for k factors is N = k^2^ + k + 1. A total number of 15 experiments including three replicates at the center field [24].

#### 3.3.2. Experimental Field

The studied factors were the initial concentration of boron, the pH of the feed compartment and the concentration of counter-ions in the receiver compartment. The limits of these factors were fixed according to the preliminary study (Section 3.1). It is generally preferable to choose variables with important effects as the variable with maximum levels to obtain maximum information from the system. In Table 4, the experimental field of studied factors is presented.

#### 3.3.3. Modelling of Donnan Dialysis

The Doehlert design is a matrix which makes it possible to estimate the coefficients of a second-order function, which is able to predict, at any point in the experimental domain, the values of the answer [24]. The chosen model describes the predicted values of the responses Y using a polynomial equation (Equation (5)).

(5) $${Y=b}_{0}{+b}_{1}X_{1}{+b}_{2}X_{2}{+b}_{3}X_{3}{+b}_{11}X_{1}^{2}{+b}_{22}X_{2}^{2}{+b}_{33}X_{3}^{2}{+b}_{12}X_{1}X_{2}{+b}_{13}X_{1}X_{3}{+b}_{23}X_{2}X_{3}$$

The estimation of the principal effect of the factor i are indicated as b_i_, and the estimation of the second-order effects are indicated as b_ii_; the estimation of the interactions between factor i and factor j are indicated as b_ij_, and the coded variables are indicated as X_i_.

#### 3.3.4. Validation of Models

The validation of models were evaluated according to two criteria: the regression coefficient (R^2^) and the percentage absolute error of deviation (AED) between the experimental and calculated results. The AED was calculated from Equation (6):

(6) $$\mathrm{AED}\;\left(\%\right)=\frac{100}{N}.\left|\frac{Y_{\exp}{-Y}_{\mathrm{theo}}}{Y_{\exp}}\right|$$

where Y_exp_ and Y_theo_ are the responses obtained from experiments and from the model, respectively. N is the number of points at which measurements were carried out. A model was considered valid if R^2^ > 0.7 and AED < 10% [47].

#### 3.3.5. Optimization by Response Surface Methodology as Doehlert Design

The objective is to simultaneously optimize the levels of these variables to attain the best system performance. In this study, response surface methodology via Doehlert design was employed with the AFN membrane to optimize the boron removal. A three-variable Doehlert experimental design involving 15 experiments, including 3 replicates at the center field (Table 5), was employed for factor optimization. We note the very small relative differences between the experimental and the calculated values.

Using the experimental results from Table 5, the second-order polynomial equation was fitted to the data appropriately, and the coefficients, *p*-values, R^2^ and AED are presented in Table 6.

In Table 6, the coefficients are presented, which shows that the pH of the feed compartment had an important effect (b_2_ = 18.81) on the removal of boron. The second influencing factor was the boron concentration (b_3_ = 9.12). However, the concentration of chloride had a less important effect on the removal of boron (b_1_ = 3.01).

The most important interaction was between the pH of the feed compartment and boron concentration (b_23_ = –12.20) which had a negative effect on the removal of boron by Donnan dialysis. However, the interaction between chloride concentration and pH of feed compartment (b_12_= –2.38) and the interaction concentration and boron concentration (b_13_= −0.10) were insignificant and had a negative effect on the removal of boron by Donnan dialysis.

The validation of the model was evaluated according to the regression coefficient (R^2^) and the percentage of absolute errors of deviation (AED), and as indicated in Table 6, the regression coefficient R^2^ = 0.999 was greater than 0.7 and the percentage absolute error of deviation AED (%) = 0.425% was less than 10%.

Analysis of variance (ANOVA) was performed. In this regard, the results are presented in Table 7. The *p*-value is defined as the ratio of the mean square effect, and the F-ratio is defined as the mean square error. For determining the effects which are statistically significant, the *p*-value has been used. The *p*-value is very important because it is near to zero; this indicates that the data are significant. According to the table of Fischer, for 5% of error, 1 degree of freedom and 15 factorial tests, the Fischer value was equal to 4.54. It seems that all the effects were significant because all their value were higher than 4.54. The Fischer value of the experimental model was much higher than the critical F value at a level of 5%. Therefore, the model is considered statistically significant. These confirm the model’s validation and confirm that the model is suitable to describe boron removal.

**Figure 6:**
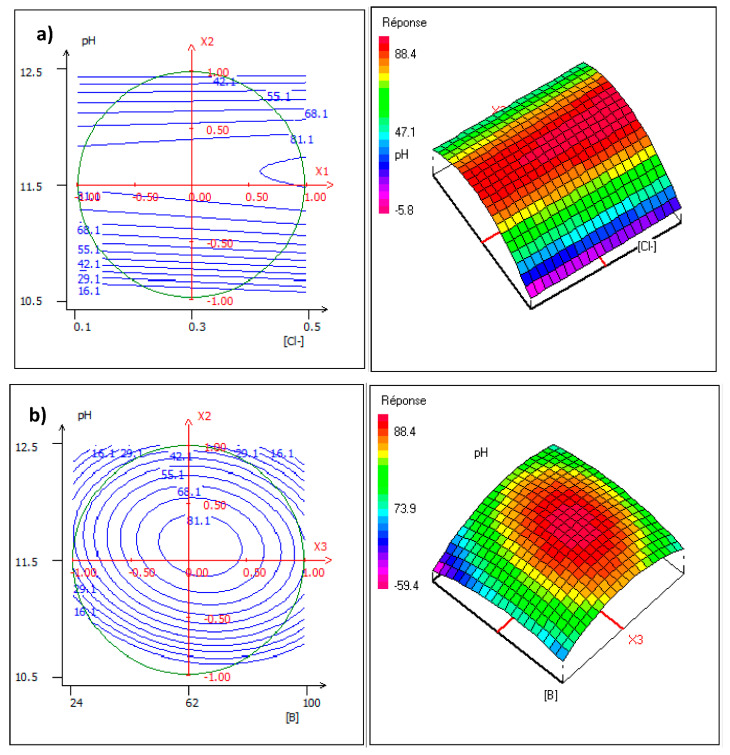

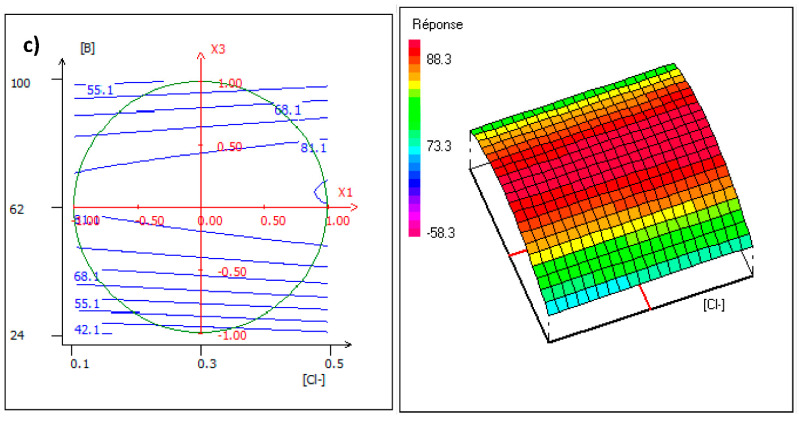
Contour plots and three-dimensional plots of (**a**) chloride concentration versus pH, (**b**) of boron concentration versus pH, (**c**) chloride concentration versus boron concentration.

In order to explain the removal of the boron by Donnan dialysis, the contour plots (curve of constant response) were used. These contour plots are a graphic analysis of iso-response curves at the chosen experimental field delimited by a circle, which confirmed the preceding results of the factorial design ((Section 3.2)). The obtained plots are provided in Figure 6.

Figure 6a shows the combined variation of chloride concentration and pH at a constant boron concentration of 62 mg/L. The shape of the contour plots shows that only around pH 11.5 did boron removal improve with an increasing chloride concentration from 0.1 mg/L to 0.3 mg/L. This was attributed to the existing forms of boron in aqueous solution under different pH values. ${B(\mathrm{OH})}_{4}^{-}$ is the dominant species at higher pH values. However, above pH 11.5, the transport of boron is probably affected by the presence and the competition with OH^−^, which decrease the removal of boron. Thus, it can be concluded that the highest boron transport was achieved at pH 11.5. Figure 6b presents the variation between the concentration of boron and pH at a constant chloride concentration of 0.3 mol/L. This result confirms that the pH greatly affects the removal of boron; this is reflected in the shape of these iso-response curves, which are concentrated in the center of the domain. This was expected because the pH and the chloride concentration were the factors which had a positive effect on the removal of boron. Figure 6c shows the variation in chloride concentration and boron concentration at a constant pH of 11.5. The shape of the contour plots show that only around a boron concentration of 62 mg/L did its removal improve when increasing the chloride concentration from 0.1 mg/L to 0.3 mg/L.

In the software NEMRODW^®^, a function named “desirability” provided the optimum value. Therefore, the optimum values were 66 mg/L for the concentration of boron, 0.5 mol/L for the concentration of chloride, and 11.6 for the pH of the feed compartment. These conditions led to a maximum boron removal of 88.8%. Three replicates of the experiment were conducted in the optimum conditions, in order to verify the efficiency of the predicted values. The coefficients of repeatability and of reproducibility were less than 1% and 5%, respectively; therefore, it can be concluded that the removal of boron by Donnan dialysis is reproducible.

Table 8 presents a comparison with previous studies of the removal of boron by Donnan dialysis. It can be concluded that the use of response surface methodology can be considered a good solution to determine the optimum conditions.

## 4. Conclusions

The removal of boron by Donnan dialysis was performed with two membranes, AFN and ACS, according to different parameters such as the pH value of the feed compartment, the concentration of counter-ions in the receiver compartment, and the concentration of boron in the feed compartment. The removal of boron by Donnan dialysis reached 75% and 48% with AFN and ACS membranes, respectively; the solution of boron had an initial concentration of 100 mg/L at pH 11.5 in the feed compartment, and 0.5 mol/L of Cl^−^, the counter-ion, in the receiver compartment. A full factorial design was conducted in order to study the influence and the interaction of parameters; thus, it was concluded that the pH is the most important parameter for the removal of boron. The response surface methodology via Doehlert enabled establishment of the optimal working conditions for the removal of boron reaching 88.8% with the AFN membrane, which were: [B] = 66 mg/L, pH = 11.6 and [Cl^−^] = 0.5 mol/L. The use of response surface methodology could be considered a good solution to determine the optimum conditions for 13.8% removal compared to the traditional “one-at-a-time” technique.

## Figures and Tables

**Table 1:** Properties of the anion-exchange membrane used for the boron removal.

Membranes	Ion-Exchange Capacity (meq/g)	Water Content (%)	Thickness (μm)
Neosepta^®^ ACS	1.85	18.9	150
Neosepta^®^ AFN	3.00	47.8	120

**Table 2:** Experimental range and factor levels studied in the factorial design.

Factors	Symbol	Coded Symbol	Range and Level	
			Lower Limit	Upper Limit
Initial concentration of boron (mg/L)	[B]	X_1_	25	100
Concentration of counter-ions (mol/L)	[Cl^−^]	X_2_	0.1	0.5
pH of the solution	pH	X_3_	10.5	12.5

**Table 3:** Full factorial design matrix.

N°	[Cl^−^]	[B]	pH	[Cl^−^]	[B]	pH	Y_B_(%)_exp_	Y_B_(%)_cal_	Relative Difference (%)
1	−1	−1	−1	0.1	25	10.5	19.9	20.2	1.50
2	+1	−1	−1	0.5	25	10.5	27.7	27.4	1.09
3	−1	+1	−1	0.1	100	10.5	30.7	30.4	0.98
4	+1	+1	−1	0.5	100	10.5	36.5	36.8	0.82
5	−1	−1	+1	0.1	25	12.5	36.9	36.6	0.82
6	+1	−1	+1	0.5	25	12.5	39.9	40.2	0.75
7	−1	+1	+1	0.1	100	12.5	41.9	42.2	0.71
8	+1	+1	+1	0.5	100	12.5	45.3	45.0	0.66

**Table 4:** Experimental range and levels of the factors.

Factors	Range and Levels						
Coded Variable X_1_	−1		−0.5	0		0.5	1
Concentration of Cl^−^ (mol/L)	0.1		0.2	0.3		0.4	0.5
Coded Variable X_2_	−0.866	−0.577	−0.287	0	0.287	0.577	0.866
pH	10.6	10.9	11.2	11.5	11.8	12.1	12.4
Coded Variable X_3_	−0.816			0		0.816	
Concentration of Boron (mg/L)	31			62		93	

**Table 5:** Doehlert matrix and the obtained results.

N°	X_1_	X_2_	X_3_	[Cl^−^]	pH	[B]	Y(%)_Exp_	Y(%)_Cal_	Relative Difference (%)
1	1.0	0.000	0.000	0.5	11.5	62	87.3	87.4	0.11
2	−1.0	0.000	0.000	0.1	11.5	62	81.5	81.4	0.12
3	0.5	0.866	0.000	0.4	12.4	62	51.4	51.6	0.39
4	−0.5	−0.866	0.000	0.2	10.6	62	16.1	15.9	1.25
5	0.5	−0.866	0.000	0.4	10.6	62	21.1	20.7	1.91
6	−0.5	0.866	0.000	0.2	12.4	62	50.7	50.6	0.19
7	0.5	0.287	0.816	0.4	11.8	93	65.2	64.9	0.47
8	−0.5	−0.287	−0.816	0.2	11.2	31	35.9	36.2	0.83
9	0.5	−0.287	−0.816	0.4	11.2	31	40.1	39.9	0.50
10	0.0	0.577	−0.816	0.3	12.1	31	46.6	46.5	0.21
11	−0.5	0.287	0.816	0.2	11.8	93	62.6	62.7	0.16
12	0.0	−0.577	0.816	0.3	10.9	93	39.5	39.6	0.25
13	0.0	0.000	0.000	0.3	11.5	62	84.2	84.2	0.00
14	0.0	0.000	0.000	0.3	11.5	62	84.2	84.2	0.00
15	0.0	0.000	0.000	0.3	11.5	62	84.2	84.2	0.00

**Table 6:** Model constants, *p*-values, R^2^ and AED values.

Coefficients		*p*-Values
b_0_	84.2	0.0001
b_1_	3.01	0.0001
b_2_	18.81	0.0001
b_3_	9.12	0.0001
b_11_	0.20	0.394
b_22_	−65.90	0.0001
b_33_	−37.40	0.0001
b_12_	−2.38	0.002
b_13_	−0.10	0.750
b_23_	−12.20	0.0001
R^2^	0.9999	
AED (%)	0.425	

**Table 7:** Analysis of variance.

Source Model	Degrees of Freedom	Sum of Square	Mean of Square	F-Value	F_table_ (α = 5%)	*p*-Value
Regression	9	7988.2	887.6	88,758.7	4.54	0.0001
Residual	5	389.1	129.7			
Total	14	8377.4				

**Table 8:** Comparative studies of boron removal by Donnan dialysis.

Reference	Composition of Feed and Receiver Compartment	Efficiency for Boron Removal (%)
[40]	Feed: 0.1 mol/L of B pH = 9.5 Receiver: 0.1 mol/L of Cl^−^	0.1% removal with AHA membrane 0.1% removal with AMH membrane 0.5% removal with AFN membrane
[18]	Feed: 75 mg/L of B pH = 11.1 Receiver: 1.0 mol/L of OH^−^	0.8% removal with AMX membrane
[19]	Feed: 20 mg/L of B pH = 9.5 Receiver: 1 mol/L of Cl^−^	30% removal by PEI−2 membrane 40% removal by PEI-3 membrane
Our work	Feed: 66 mg/L of B pH = 11.5 Receiver: 0.5 mol/L of Cl^−^	88.8% removal by AFN membrane

## Data Availability

Not applicable.
